# Benign Acute Myositis in Children: A Case Series

**DOI:** 10.7759/cureus.73303

**Published:** 2024-11-08

**Authors:** Raja Arrab, Youssef Benchehab, Insaf Alaammari, Nezha Dini

**Affiliations:** 1 Paediatrics, Mohammed VI International University Hospital, Mohammed VI University of Health Sciences (UM6SS), Casablanca, MAR; 2 Paediatrics, Mohammed VI University of Health Sciences (UM6SS), Casablanca, MAR; 3 Paediatrics, Cheikh Khalifa International University Hospital, Mohammed VI University of Health Sciences (UM6SS), Casablanca, MAR; 4 Paediatrics, Cheikh Khalifa International University Hospital, Mohammed V University, Faculty of Medicine and Pharmacy of Rabat, Casablanca, MAR

**Keywords:** benign acute myositis, children, creatine phosphokinase, influenza, myoglobinuria

## Abstract

Benign acute myositis is a rare and transient condition that usually occurs after a viral upper respiratory infection. Several viruses have been associated with this process particularly influenza B. Viral myositis frequently occurs in school-age children with a male predominance. Patients complain about symmetrical calf muscle pain and uncoordinated gait and functional impairment. The presentation is associated with increased creatine phosphokinase. Benign acute myositis is a self-limiting condition with an excellent prognosis. Myoglobinuria is uncommon but severe. We report a case series of three patients presented with viral myositis. Pediatricians must be aware of this pathology to reach an early diagnosis and avoid unnecessary diagnosis investigations. It is also important to reach out to myoglobinuria, which can lead to renal impairment.

## Introduction

Benign acute myositis is a rare muscle disorder that primarily affects previously healthy children [1], typically following an upper respiratory viral infection [2].

It is frequently associated with influenza infection [3] and affects preschool and school-age children [2]. It is less prevalent among adults [4]. It is characterized by an acute difficulty to walk with symmetrical calf pain. The most common laboratory finding is the elevated creatine phosphokinase (CPK) level. While the exact pathogenesis remains unclear, the condition is generally self-limiting and non-severe [4]. However, in rare instances, complications such as rhabdomyolysis may arise, posing a risk to renal function [1].

In this case series, we present three pediatric patients diagnosed with benign acute viral myositis, highlighting their clinical presentations and outcomes.

## Case presentation

Case 1

A 13-year-old boy presented to the pediatric emergency department with sudden-onset functional impairment of the lower limbs. The patient had a fever and a history of cough and sore throat over the past six days. His parents had administered antibiotics (amoxicillin-clavulanic acid) and ibuprofen prior to the visit.

On clinical examination, the patient was alert and febrile, with a temperature of 38.8°C. Cardiovascular examination revealed tachycardia at 100 beats per minute, but no other auscultatory abnormalities. Neurologically, the patient showed normal tendon reflexes and no sensory deficits. However, he exhibited functional weakness in both lower limbs accompanied by symmetrical calf pain. Pharyngitis was noted on examination, with clean eardrums and no skin rash. A urinary dipstick test was negative. The remainder of the physical examination was unremarkable.

Laboratory tests revealed normal blood count values. C-reactive protein was negative at 0.2 mg/L. Notably, muscle enzymes were elevated, with a CPK level of 520 IU/L and lactate dehydrogenase (LDH) at 299 IU/L (Table 1).

**Table 1:** Case 1 laboratory investigations

	Day 1	Day 7	Normal range
Hemoglobin	15.1 g/dL	-	11.1-14.7 g/dL
White blood cell count	3320/mm ^3^	-	4000-14,500/mm^3^
Neutrophils	1000/mm^3^	-	1500-8000/mm^3^
Lymphocytes	2060/mm^3^	-	1000-7000/mm^3^
Monocytes.	270/mm^3^	-	150-1300/mm^3^
Platelets	170,000/mm^3^	-	166,000-463,000/mm^3^
C-reactive protein	0.2 mg/L	-	0.1-2.8 mg/L
Creatine phosphokinase (CPK)	520 UI/L	76 UI/L	< 200 UI
Lactate dehydrogenase (LDH)	299 UI/L	206 UI/l	85-230 UI/l

The patient was diagnosed with acute viral myositis. Given his overall condition, hospitalization was deemed necessary, and he received symptomatic treatment, including intravenous hydration, analgesics, and vitamin supplementation. He was discharged after three days, with significant improvement in his physical condition.

Case 2

An 11-year-old previously healthy child presented to the pediatric emergency department with acute fever. The initial diagnosis was tonsillitis, and the patient was started on oral antibiotics. However, two days later, the fever persisted, and the child developed difficulty walking due to sudden weakness.

On clinical examination, the patient was conscious and febrile. He showed no signs of nuchal rigidity. Cardiovacular and respiratory examination revealed no auscultatory abnormalities. Neurological assessment showed symmetrical calf muscle pain and an uncoordinated gait. The tendon reflexes were normal. There was no sensory deficits. The rest of the physical examination was unremarkable aside from the tonsillitis. A urinary dipstick test was negative.

Laboratory investigations revealed white blood cell count at 2,600/mm^3^ with lymphocytes at 900/mm^3^. CRP was negative at 0.8 mg/L, and CPK levels were significantly elevated at 1,889 IU/L (Table 2).

**Table 2:** Laboratory investigations of Case 2

	Day 1	Day 7	Normal range
Hemoglobin	13.5 g/dL	13.3 g/dL	11.1-14.7 g/dL
White blood cell count	2600/mm^3^	4860/mm^3^	4000-14,500/mm^3^
Neutrophils,	1400/mm^3^	2000/mm^3^	1500-8000/mm^3^
Lymphocytes	900/mm^3^	2340/mm^3^	1000-7000/mm^3^
Monocytes	310/mm^3^	490/mm^3^	150-1300/mm^3^
Platelets	223,000/mm^3^	265,000/mm^3^	166,000-463,000/mm^3^
C-reactive protein	0.8 mg/L	0.2 mg/L	0.1-2.8 mg/L
Creatine phosphokinase (CPK)	1889 UI/L	200 UI/l	< 200 UI

The patient was admitted for observation and received intravenous hydration, antibiotics, and analgesics. After three days of treatment, the patient showed clinical improvement and was discharged.

Case 3

A previously healthy five-year-old child presented to the pediatric emergency department with fever, abdominal pain, and vomiting. Physical examination revealed pharyngitis, and an abdominal ultrasound showed mesenteric lymphadenopathy. Blood tests revealed a white blood cell count of 3,660/mm³ with lymphopenia at 730/mm³. CRP was negative at 1 mg/L. The child was initially started on oral analgesics and antibiotics.

One week later, the patient developed bilateral calf pain and an uncoordinated gait. On clinical examination, muscle strength and tendon reflexes were normal, and no sensory deficits were noted. However, the child reported symmetrical pain in the quadriceps and calf muscles. There was no skin rash. The cardiovascular and pulmonary examinations were normal. A urinary dipstick test was negative.

The biological assessment showed elevated muscle enzymes, with CPK at 1,234 IU/L and LDH at 432 IU/L (Table 3).

**Table 3:** Laboratory investigations of Case 3

	Day 1	Day 7	Normal range
Hemoglobin	12.8 g/dL	-	11.1-14.7 g/dL
White blood cell count	3660/mm^3^	-	4000-14500/mm^3^
Neutrophils	2400/mm^3^	-	1500-8000/mm^3^
Lymphocytes	730/mm^3^	-	1000-7000/mm^3^
Monocytes	510/mm^3^	-	150-1300/mm^3^
Platelets	170,000	-	166,000-463,000/mm^3^
C-reactive protein	1 mg/L	-	0.1-2.8 mg/L
Creatine phosphokinase (CPK)	1234 UI/L	191 UI/L	< 200 UI
Lactate dehydrogenase (LDH)	432 UI/L	411 UI/L	85-230 UI/L

The patient was treated with oral analgesics, hydration, and vitamin supplementation. He was discharged home and showed clinical and biochemical improvement within one week. A follow-up assessment revealed a significant decrease in CPK levels to 191 IU/L.

## Discussion

Benign acute myositis, also known as influenza myositis, is a rare muscle disorder. It was first described by Leichtenstern in 1905, and in 1957, Lunderberg reported the clinical presentation in school-age children during an influenza epidemic, coining the term "myalgia cruris epidemica" [1,2].

The exact pathogenesis of benign acute myositis remains unclear. It may result from either direct viral infection of muscle cells or an immune-mediated mechanism [3]. Experimental studies involving the influenza virus have suggested increased viral tropism for immature muscle cells, which may explain its higher prevalence in children [4]. It has also been hypothesized that the virus acts as a trigger, especially in genetically predisposed children or those with underlying metabolic disorders [4].

The prevalence of benign acute myositis is not well established but is predominantly seen in school-age children, with rare cases reported in adults [4]. Its epidemic nature has been documented, with most cases appearing in clusters during influenza outbreaks [2]. Myositis can occur either during the active phase of influenza or, more commonly, during the convalescent period. A strong male predominance has been noted in most studies, with a male-to-female ratio ranging from 2:1 to 4:1, likely due to genetic factors [5-7].

Benign acute myositis is most frequently associated with influenza A and B viruses, though other viral agents have been implicated, including echoviruses, enteroviruses (particularly coxsackie virus), parvovirus B19, hepatitis E virus, and respiratory syncytial virus [8]. Mycoplasma pneumoniae has also been linked to myositis [8,9]. In our study, the diagnosis was based on clinical presentation - functional weakness following an upper respiratory infection - and elevated CPK levels. Molecular diagnosis using multiplex PCR was not performed.

The clinical presentation of benign acute myositis is characteristic [5]. Following an episode of influenza, children typically complain of symmetrical calf pain, difficulty or refusal to walk, and an uncoordinated gait. They may maintain their feet in plantar flexion to alleviate discomfort [7]. Our patients' reported signs are in line with the clinical presentation in the literature.

A thorough medical history should assess for neuromuscular diseases, medication use, metabolic or thyroid disorders [1] and signs of upper respiratory infections (e.g., rhinorrhea, fever, cough) in the days preceding symptom onset. In our study, clinical signs of myositis were present within two days to one week after an upper respiratory infection.

In the majority of cases, general health remains preserved, and there are no abnormalities on neurological examination. The diagnosis is generally straightforward, particularly in a school-age boy following a viral infection. However, other conditions presenting with similar symptoms, such as fractures, Guillain-Barré syndrome, arthritis, osteomyelitis, transverse myelitis, cerebellar ataxia, juvenile idiopathic arthritis, muscular dystrophy, or dermatomyositis, must be ruled out [1,10,11].

Influenza-associated myalgia is a milder condition that occurs concurrently with the influenza infection and is characterized by normal CPK levels [11].

For pediatricians, a rigorous clinical approach is essential: the timing of symptoms post-viral infection, symmetrical calf pain, a normal neurological exam, the absence of skin changes, and elevated CPK levels are generally sufficient for diagnosis [10].

Although benign acute myositis is generally self-limiting, complications can occur. Myocarditis, for instance, is more frequently reported in adults [8]. In some cases, rhabdomyolysis may develop, leading to myoglobinuria and, potentially, acute tubular necrosis [12]. While this complication is rare in children and tends to have a better prognosis compared to adults [2], it should be considered if the condition does not resolve spontaneously. It is highly recommended to perform a urine dipstick test to screen for myoglobinuria. None of the patients in our study exhibited abnormalities on urine dipstick tests, and renal function remained normal in all cases.

When the clinical presentation is typical, further investigations are generally unnecessary beyond measuring CPK levels and performing basic inflammatory tests [8]. In our study, the most laboratory finding was the elevation of CPK levels; its level ranged from 520 to 1889 UI/L. Similar results were reported in the study of Rubin et al. [4]. Other studies showed more elevated levels of CPK [5,7]. The decrease in CPK levels was obtained during the second week of control, consistent with the literature [4].

Regarding the blood count, lymphopenia was noted in two patients. Leukopenia (including neutropenia and/or lymphopenia)was reported in 60% of the literature [5,7]. Studies showed 30% of normal blood count [7] similar to our study.

Muscle biopsy is not required for diagnosis and, if performed, would likely show either normal results or areas of necrosis with inflammatory infiltrates [8].

The management of benign acute myositis in children does not require specific treatment [9]. Symptomatic measures, such as rest, hydration, and analgesics, are typically sufficient for rapid recovery [5,10,11]. Hospitalization is not usually needed unless the patient’s general condition worsens or they are unable to take oral medications at home [1,11]. In our study, two patients were hospitalized due to deteriorating general health and their inability to tolerate oral treatment at home.

Benign acute myositis is a self-limiting condition, with most patients recovering spontaneously within a few days with symptomatic care. In one study, the prognosis appeared better in patients who tested positive for influenza [13], although antiviral treatment with oseltamivir did not seem to influence the course of the disease [13].

Influenza vaccination plays a crucial role in preventing influenza-related complications. However, its effectiveness in preventing acute viral myositis remains unproven [9]. None of the patients in our study had received the influenza vaccine.

## Conclusions

Acute viral myositis is a benign condition that is typically easily recognizable with a careful and systematic clinical evaluation. A rigorous approach enables pediatricians to make an early diagnosis, efficiently exclude more severe conditions, and avoid unnecessary diagnostic procedures.

Though viral myositis is generally self-limiting and can be effectively managed with symptomatic measures in most cases, rare complications may occur. Therefore, a urine dipstick test is essential for detecting potential myoglobinuria, which could compromise renal function.
